# How Specificity in Episodic Future Thinking Affects Prospective Memory: Cognitive Mechanisms and Latent Subgroup Differences

**DOI:** 10.3390/bs16040546

**Published:** 2026-04-06

**Authors:** Chen Cai, Zihan Quan, Qingye Lin, Xin Fang, Qiyu Lin

**Affiliations:** 1 Department of Psychology, School of Education Science, Qingdao University, Qingdao 266071, China; linqy040623@qdu.edu.cn (Q.L.); fangxin@qdu.edu.cn (X.F.); 2 School of Psychology, South China Normal University, Guangzhou 510631, China; hannah_quan@163.com (Z.Q.); 2025023717@m.scnu.edu.cn (Q.L.)

**Keywords:** prospective memory, episodic future thinking, constructive retrieval hypothesis, dynamic multiprocess framework, latent profile analysis

## Abstract

Episodic future thinking (EFT) has been confirmed as a promising cognitive intervention for enhancing prospective memory (PM), yet emerging evidence suggests its effects may depend on the specificity of induction. The current study investigated this issue by dichotomizing EFT into two distinct methods: specific (researcher-guided detailed mental simulations) versus non-specific (participants’ self-guided imagination), implemented through differentially structured future thinking instructions. We also analyzed the distinct cognitive strategies mainly employed under each EFT condition based on the *Dynamic Multiprocess Framework*. The latent profile analysis (LPA) was further conducted to characterize individual variability in responsiveness to EFT manipulations. Behavioral results revealed comparable PM accuracy improvements across both EFT methods relative to the control group; moreover, specific EFT uniquely accelerated response times for both PM and ongoing task execution. The LPA further identified three distinct EFT response patterns—self-competent, proactive, and reactive—each exhibiting unique state-dependent cognitive characteristics. These findings provide a refined understanding of the EFT-PM relationship: (1) specific EFT facilitates more automatic retrieval of PM intentions, whereas non-specific EFT predominantly engages strategic monitoring; (2) individual differences in baseline mental images influence the effectiveness of EFT methods, suggesting the potential benefits of personalized intervention approaches for PM enhancement.

## 1. Introduction

In daily life, we plan and organize upcoming tasks constantly, from remembering to purchase a cup of coffee while passing by Starbucks to attending a business meeting at 3:00 p.m. This ability to remember and effectively carry out an intended action in the future is known as prospective memory (PM), which plays a crucial role in our daily functioning and allows us to successfully complete routine tasks (M. Kliegel et al., 2001; Walter & Meier, 2014). The multi-phase process of PM comprises four distinct yet successive stages: intention formation, retention, initiation, and execution (M. Kliegel et al., 2011; M. Kliegel et al., 2000). This framework highlights the importance of effectively encoding PM intentions, as this process supports successful performance in later stages of prospective memory.

Episodic future thinking (EFT) is a higher-order cognitive process that enables individuals to project themselves into the future through mental simulation, and then pre-experience future events in advance (Atance & O’Neill, 2001; Faustmann & Altgassen, 2024). For example, a person might imagine arriving at a supermarket after work, noticing the pharmacy counter, and remembering to collect a prescription. Such future-oriented simulations render intended actions more vivid and contextually grounded, thereby facilitating the execution of upcoming tasks. Many studies have confirmed that EFT is an effective encoding strategy which can clearly boost PM abilities (Altgassen et al., 2016; Altgassen et al., 2015; Cottini et al., 2021). With EFT employment, individuals can immerse themselves in the scenarios where they execute prospective events, thereby enhancing their encoding of contents of tasks. The encoding details, stored in retrospective memory as a crucial component of PM intentions, can finally contribute to the PM performance (Altgassen et al., 2014). Specifically, with a vivid pre-encoding of the detailed progression of PM tasks, participants demonstrated prolonged retention, improved initiation and execution of PM intentions (Neroni et al., 2014). These findings clearly manifest the potential of EFT method as a valuable tool to improve PM abilities.

In previous studies, EFT has typically been implemented in two ways: either through researcher-guided instructions or via participants’ spontaneous imagination of future scenarios (Hollis-Hansen et al., 2019; Kretschmer et al., 2019). The former, referred to as specific EFT approach, involves researchers providing participants with detailed guidance on how to envision performing the PM task. In contrast, the latter asks participants to generate future imagery autonomously based on general experimental instructions, termed the non-specific EFT approach. This distinction is potentially critical because, according to the *Constructive Retrieval Hypothesis*, instructions that direct attention to episodic details (i.e., episodic specificity induction) augment the richness and vividness of imagined future events (Madore et al., 2019). Moreover, research suggests that greater vividness in mental imagery promotes more efficient retrieval of stored information (Abel et al., 2024; Pylyshyn, 2002). Together, these findings indicate that different EFT maneuvers may lead to varying levels of specificity and vividness during future event simulation. In particular, EFT instructions that emphasize contextual details could facilitate faster retrieval of PM intentions at the time of task execution.

**Hypothesis** **1.** *Both EFT methods enhance PM performance, but with differential efficacy. Specifically, specific EFT will yield greater PM performance improvements than non-specific EFT, which will be manifested in higher accuracy and shorter response time in PM task.*

The *Dynamic Multiprocess Framework* states that individuals employ two primary cognitive strategies to retrieve PM cues during task performance: automatic retrieval or strategic monitoring (Scullin et al., 2013). Automatic retrieval is typically activated by external cues, which can directly trigger the recall of a pre-stored intention. This process generally occurs without conscious effort and consumes minimal cognitive resources. Conversely, strategic monitoring requires individuals to deliberately allocate enough resources to actively search for PM cues while engaged in an ongoing task, which imposes substantial demands on attentional capacity. In the process of PM task execution, participants’ selection of cognitive strategies can be flexibly adjusted in response to factors such as environmental cues, task complexity, and metacognitive processes (Gan et al., 2024; Meier et al., 2006). This flexibility is mainly reflected in the response time of the ongoing task (Shelton & Scullin, 2017). Specifically, prolonged response times are widely interpreted as indicative of strategic monitoring engagement, wherein cognitive resources are diverted to sustain PM intentions, which inevitably compromises ongoing task performance.

Prior research examining the cognitive mechanisms of EFT in PM tasks has often compared ongoing task accuracy between EFT and control conditions (Ma & Zhang, 2024; McFarland & Glisky, 2012). They report comparable ongoing task performance across groups, suggesting that EFT enhances PM without interfering with concurrent tasks. This pattern has been interpreted as evidence that EFT strengthens cue–action associations during encoding, which reduces reliance on effortful monitoring and promotes automatic retrieval during execution. However, interpretations based solely on ongoing task accuracy has limitations, particularly within the *Dynamic Multiprocess Framework*. Moreover, given the distinct manipulations of EFT, it is necessary to reexamine their underlying mechanisms separately, particularly with regard to response time metrics. Considering that specific EFT facilitates encoding through vivid episodic simulation (Gjorgieva et al., 2023; Marre et al., 2021), primarily by establishing stronger cue-action linkages, it reduces the necessity for sustained strategic monitoring and promotes a transition to automatic retrieval during task execution (Einstein & McDaniel, 2005). In contrast, non-specific EFT involves relatively shallow encoding, which might impose higher cognitive load for PM intention retrieval. Such heightened demands may consequently impair ongoing task performance. Nonetheless, this proposition warrants further empirical investigation.

**Hypothesis** **2.** *Specific EFT will elicit more automatic retrieval during PM tasks, whereas non-specific EFT will induce greater reliance on strategic monitoring. This difference can be manifested by a shorter response time for the ongoing task in the specific EFT condition compared to the non-specific EFT condition.*

Previous studies mainly adopted a variable-centered approach to investigate the effects of EFT on PM performance by manipulating EFT as an experimental condition (Cheng et al., 2021; Cottini et al., 2021; Faustmann & Altgassen, 2024; Ma & Zhang, 2024). In such designs, participants were typically assigned to different encoding conditions, and their subsequent PM performance was compared at the group level. While these studies have advanced our understanding of the overall effectiveness of EFT, they have largely overlooked a potentially critical issue: individuals may not respond uniformly to EFT interventions. Variations in receptivity, engagement, or responsiveness to EFT across individuals may meaningfully shape its effects on PM performance. To better capture such heterogeneity, the present study introduces latent profile analysis (LPA) as a supplementary person-centered analytical approach. LPA identifies distinct latent subgroups of individuals based on shared response patterns across observed indicators (Gabriel et al., 2018; M. C. Howard & Hoffman, 2018; Spurk et al., 2020). This method offers an advantage over traditional group-based analyses by revealing whether qualitatively different response profiles exist within the sample, rather than assuming a uniform effect of EFT across participants. In addition, by examining subgroup differences within a unified analytical framework, LPA reduces reliance on multiple separate comparisons and helps mitigate the risk of Type I error inflation (Lanza & Rhoades, 2013). Thus, incorporating LPA allows the current study to go beyond estimating the average effect of EFT and to examine systematic individual differences in how participants benefit from EFT interventions.

## 2. Methods

### 2.1. Participants

A prior sample size estimation using G*Power 3.1.9.7 (Faul et al., 2007) determined a minimum requirement of 111 participants for the current study, with a medium effect size (f = 0.30) under an α level of 0.05 and the power of 0.8. A total of 126 participants were initially recruited for the experiment (57 males; mean age = 18.87 ± 1.09 years) and randomly assigned to one group. Three participants were excluded during manipulation check procedures due to non-compliance with EFT instructions. No age differences were found among the three groups, *p* = 0.407. None of the participants had previously undergone similar cognitive testing. All met inclusion criteria of right-handedness and normal or corrected-to-normal visual acuity, and provided written informed consent in accordance with the Declaration of Helsinki. The experiment was approved by the Institutional Review Board of Psychology of Qingdao University (*IRB No. QDU202406210012*) and the participants would receive 0.5 course credits as compensation.

### 2.2. Design

The current experiment employed a one-factor three-level (group: specific EFT vs. non-specific EFT vs. control) between-subjects design. The EFT manipulations were adapted from the paradigm developed by Ma and Zhang’s (2024) and varied in the degree of specificity provided in the instructions. Participants were randomly assigned to one of the following three conditions: (1) the specific EFT group imagined future events by listening to a prerecorded auditory instruction for two minutes, which detailed the task execution sequence, participants’ anticipated affective states, and their context-dependent behavioral responses. This standardized procedure ensured consistency across participants and minimized potential experimenter bias. Then participants elaborated their mental simulation for three minutes, resulting in a total EFT duration of five minutes; (2) the non-specific EFT group were asked to independently generate task-relevant imagination for two minutes, followed by a three-minute description of their imagined contents. The entire process also lasted for about five minutes; (3) the control group received standard task instructions devoid of any mental simulation components. The dependent variables included PM performance, indexed by accuracy (ACC) and response time (RT), and imagery ratings, comprising both participant self-assessments as well as rater assessments.

To ensure protocol fidelity and validate experimental manipulations, a comprehensive triangulated assessment approach was implemented. Participants completed self-rating before and after the imagery task using a 7-point Likert scale (1 = no vivid imagery, 7 = perfectly vivid) to evaluate their mental simulations. Concurrently, three independent raters, who were blinded to experimental conditions, were recruited to objectively evaluate the quality of participants’ audio-recorded simulation descriptions. All raters underwent systematic and standardized training prior to scoring. They assessed each response based on two dimensions: procedural specificity (i.e., accuracy of the task sequence) and contextual embodiment (i.e., vividness of the future scenario), each rated on a 7-point scale. The maximum attainable score of 14 points per rater was aggregated across raters, which demonstrated excellent interrater reliability (Cronbach’s α = 0.886).

### 2.3. Materials

#### 2.3.1. Emotional Words

Fifty emotional words, half positive and half negative, were systematically selected from the Chinese Affective Words System (Luo & Wang, 2004), matched across *Pleasure*, *Arousal*, *Familiarity*, and *Dominance* dimensions. Word frequency was further cross-validated using the Modern Chinese Frequency Dictionary (Wang et al., 1986). Four of these words, balanced on valence, served as PM targets: two positive words “文雅” (elegant) and “热烈” (enthusiastic) and two negative words “鲁莽” (reckless) and “枯燥” (boring). An additional 12 words were used as practice stimuli, yielding a total of 62 words being displayed in the experiment. Moreover, we ensured that the emotional words differed exclusively on the *Pleasure* dimension (*t*(52.980) = 42.732, *p* < 0.001, *d* = 10.850, 95%CI = [3.22, 3.53]), while remaining equivalent on *Arousal*, *Familiarity*, *Dominance*, word frequency, and stroke count (*p*s = 0.085~0.894). The experiment was conducted on a computer equipped with E-Prime 3.0 software, with each word presented three times to participants in a randomized order.

#### 2.3.2. Vividness of Visual Imagery Questionnaire (VVIQ)

The VVIQ was administered to quantify individual differences in mental simulation capacity (Marks, 1973). It consists of two subsections measuring visualization ability under eyes-open and eyes-closed conditions, each encompassing 16 standardized scenarios. Participants were instructed to imagine each scenario and then rate their imagery clarity on a 5-point scale, with higher scores indicating greater vividness. The imagery score was calculated by averaging responses across all 32 items. The VVIQ demonstrated excellent internal consistency in the present sample (Cronbach’s α = 0.918). Moreover, baseline imagery capacity did not differ significantly across the three groups (specific EFT group: *M* = 3.67, *SD* = 0.52; non-specific EFT group: *M* = 3.74, *SD* = 0.52; control group: *M* = 3.56, *SD* = 0.50, *p* = 0.304).

### 2.4. Experimental Procedures

**Figure 1:**
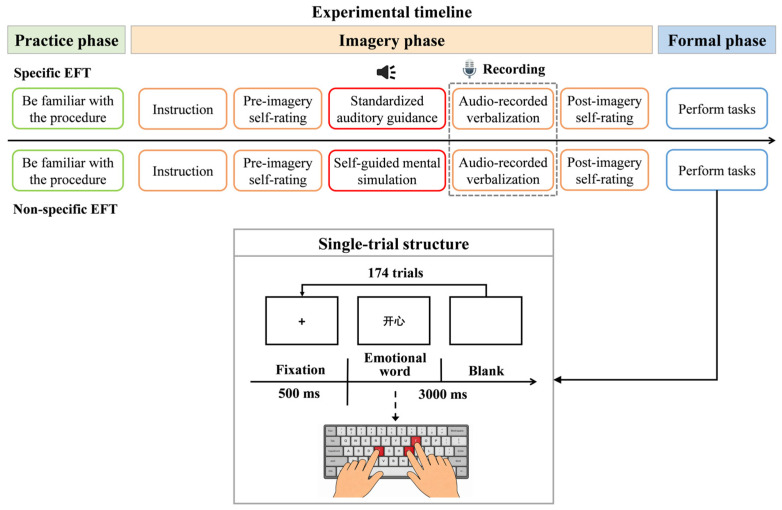
The experiment’s procedure for specific and non-specific EFT groups. (**Upper panel**)**:** Schematic of the full experimental sequence. The red boxes highlight the critical difference between the two EFT conditions during the imagery phase: the specific EFT group received standardized auditory guidance, whereas the non-specific EFT group engaged in self-guided mental simulation. (**Lower panel**)**:** Structure of a single trial. The emotional word “开心” (meaning “Happy”) is shown here as an example of a positive-valence stimulus, for which the correct ongoing task response was to press the “F” key. Negative words required a “J” key press. When a predefined PM target word appeared, participants only needed to press the “I” key.

To begin with, all participants were randomly assigned to one of the three groups and instructed to fill out the VVIQ. Then they had to go through a three-phase experimental protocol stated as below (see Figure 1).

Practice phase. In this initial phase, participants were asked to complete a computerized dual-task paradigm consisting of 36 trials. The ongoing task was judging the valence of words (pressing “F” for positive words and “J” for negative words) and the PM task was to press “I” whenever the target words appeared. Each trial began with a 500 ms central fixation cross, followed by the presentation of an emotional word, which was terminated upon response. The total duration of the word stimulus and the subsequent interstimulus interval was 3000 ms, with the interval dynamically adjusted to participants’ response speed to the preceding stimulus.

Imagery phase. Participants first conducted a three-minute arithmetic distractor task to wipe off working memory residuals. They then received instructions for the upcoming experimental phase, which mirrored the practice phase except for the PM target words involved. Thereafter, they completed a pre-imagery self-rating on vividness before enrolling in different EFT conditions. The specific EFT group listened to a standardized auditory recording that guided them through a detailed simulation of task performance. After that, they were asked to articulate their visualizing details, which were audio-recorded for subsequent analysis with their informed consent. In the end, they had to finish a post-imagery self-rating. In contrast, the non-specific EFT group independently generated a mental simulation of the task and likewise described it aloud for recording, followed by the post-imagery self-rating. The control group bypassed simulation procedures and proceeded directly to the next phase.

Formal phase. In this phase, 174 trials equally divided into two blocks were executed. The procedure was identical to the practice phase, except that new word stimuli and PM targets were introduced. This implementation maintained task familiarity while introducing necessary novelty to uphold ecological validity across all experimental groups.

### 2.5. Statistical Analysis

For rater-rating scores received by the participants, a total score below 8 (with the median of 4 as the cutoff per dimension) was considered indicative of ineffective EFT implementations. Based on this criterion, 10 participants were excluded, leaving 113 participants (see Table 1; 54 males; mean age = 18.75 years, *SD* = 0.892) for subsequent analysis. For participants’ dual task performance, we first trimmed trials in which RTs fell out of three standard deviations from the group means within correct trials for each group. Then we adopted one-way ANOVAs with a significance level of 0.05 to compare group differences in ACCs and RTs for both tasks. To analyze the imagery rating scores of two EFT groups, linear mixed models (LMMs) were fitted using restricted maximum likelihood estimation (REML), which allowed us to examine experimental effects while accounting for individual differences (R. Kliegel et al., 2010). Prior to analysis, self-rating and rater-rating scores were converted to log-transformed values to approximate normality. Two LMMs were specified: the first included group (specific vs. non-specific EFT), self-rating time (pre- vs. post-imagery), and their interaction as fixed effects, with participants modeled as random intercepts. The second model incorporated group, rating agent (self vs. raters), and their interaction as fixed effects, still with participant as random intercepts. Model parameters were interpreted through regression coefficients (*b*), standard errors (*SE*), *t*/*z*-statistics, *p*-values, and 95% confidence intervals (Cox & Hasselman, 2013). To assess model adequacy, we additionally examined convergence and singularity diagnostics, multicollinearity among fixed effects, and residual diagnostics. Specifically, convergence was assessed from optimizer output, singularity was evaluated using singular fit diagnostics, multicollinearity was examined using variance inflation factors (VIFs), and residual-versus-fitted plots and Q-Q plots were inspected to assess model assumptions.

Moreover, we performed Bayes Factor (BF) analysis using the anovaBF and lmBF functions from the *BayesFactor* package (Morey et al., 2018). In hypothesis testing, the BF quantifies the strength of evidence in the current data for the alternative hypothesis relative to the null hypothesis (Hu et al., 2018). It provides a continuous measure of the extent to which the valid data supports each hypothesis, instead of the binary null-hypothesis significance testing in traditional frequentist statistics. Following conventional guidelines (Jarosz & Wiley, 2014; Wetzels et al., 2011), BF > 1 indicates support for the alternative hypothesis, with higher values suggesting stronger power (e.g., BF > 3 indicates moderate support, BF > 10 suggests strong support).

Finally, LPA was conducted in Mplus 8.3 (Muthén & Muthén, 2017) to identify subgroups of participants exhibiting distinct patterns of responsiveness to EFT. Participants’ self-ratings from both pre- and post-imagery were used as indicators, as they directly reflect participants’ subjective experience of the EFT manipulations and allow for sensitive assessments of changes in intervention effects over time. Models with increasing numbers of latent profiles were estimated and compared sequentially. The model selection was based on: (1) information-theoretic indices, including Akaike Information Criterion (AIC), Bayesian Information Criterion (BIC), and sample-size adjusted BIC (ABIC), where lower values indicate better fit; (2) comparative fit indices, such as the Lo–Mendell–Rubin adjusted likelihood ratio test (LMR-LRT) and the bootstrap likelihood ratio test (BLRT), which can assess the significance of model fit improvement between two successive class solutions (K vs. K − 1) at α < 0.05; (3) classification quality metrics, with entropy values approaching 1 indicating greater precision in classification. To reduce the risk of local solutions, all models were estimated using 200 random starts and 50 final-stage optimizations. The final model was selected based on a combination of statistical adequacy and theoretical interpretability (J. Howard et al., 2016).

## 3. Results

### 3.1. Analysis of Task Performance

**Figure 2:**
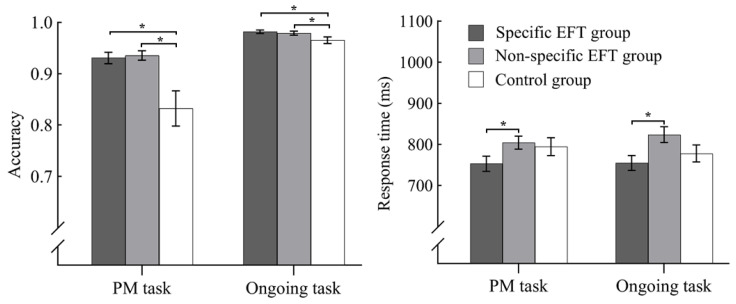
Accuracy and response time of the three groups in dual tasks. Bars represent standard errors. * *p* < 0.5.

As shown in Figure 2, the results revealed statistically reliable effects of EFT methods on PM accuracy, as evidenced by a significant univariate main effect (*F*(2, 112) = 8.314, *p* < 0.001, η^2^ = 0.131) and substantial Bayesian support (BF = 61.441). Post hoc analysis demonstrated that both specific (*M* = 0.93, *SD* = 0.07) and non-specific EFT (*M* = 0.94, *SD* = 0.06) groups outperformed the control group (*M* = 0.83, *SD* = 0.20) with moderate-to-strong evidentiary support (specific EFT vs. control: *p* = 0.028, BF = 8.885; non-specific EFT vs. control: *p* = 0.018, BF = 14.693). The two experimental groups showed equivalent ACCs (*p* = 0.983, BF = 0.244). The RT comparisons among the three groups also exhibited differences in PM task. Specifically, although the main effect of EFT methods was not significant (*p* = 0.081, BF = 0.689), the multiple comparisons showed that the specific EFT group (*M* = 753.02, *SD* = 115.54) responded significantly faster than the non-specific EFT group (*M* = 804.15, *SD* = 100.09; *p* = 0.045, BF = 1.854). No differences were found either between the control group (*M* = 794.37, *SD* = 125.86) and the specific EFT group (*p* = 0.065, BF = 0.882) or between the control group and the non-specific EFT group (*p* = 0.949, BF = 0.243).

Parallel analyses of ongoing task accuracy also yielded significant differences among the three groups (*F*(2, 112) = 3.408, *p* = 0.037, η^2^ = 0.058, BF = 1.299). Both the specific (*M* = 0.98, *SD* = 0.02) and non-specific EFT (*M* = 0.98, *SD* = 0.03) groups maintained superior performance compared to the control group (*M* = 0.97, *SD* = 0.04; specific EFT vs. control: *p* = 0.015, BF = 2.541; non-specific EFT vs. control: *p* = 0.041, BF = 1.071). No significant difference was found between the two EFT groups (*p* = 0.672, BF = 0.259). RT analysis exhibited a significant main effect of group (*F*(2, 112) = 3.737, *p* = 0.027, η^2^ = 0.064, BF = 1.786) in that the specific EFT group (M = 755.02, SD = 115.31) responded faster than the non-specific EFT group (M = 823.74, SD = 122.21; *p* = 0.008, BF = 6.071). However, no differences were detected between the control group (M = 777.68, SD = 118.55) and the specific EFT group (*p* = 0.288, BF = 0.405) or between the control group and the non-specific EFT group (*p* = 0.131, BF = 0.599).

### 3.2. Analysis of Imagery Rating Score

The results from the one-way ANOVA showed no significant differences among the three groups in pre-imagery self-rating scores, (specific EFT: *M* = 3.80, *SD* = 1.76; non-specific EFT: *M* = 4.13, *SD* = 1.56; control: *M* = 4.15, *SD* = 1.75, *p* = 0.597). Nonetheless, LMMs incorporating self-rating time indicated that the optimal model included self-rating time as the fixed effect and participants as the random effect (BF = 4.20). Parameter estimates illustrated that both specific and non-specific EFT groups manifested a significant post-imagery elevation in self-reported vividness. Specifically, the specific EFT group showed a robust increase (*b* = 0.586, *SE* = 0.091, *t* = 6.424, *p* < 0.001, 95%CI = [0.40, 0.77]), and the non-specific EFT group also showed a significant increase (*b* = 0.344, *SE* = 0.091, *t* = 3.770, *p* < 0.001, 95%CI = [0.16, 0.53]). The BF for the self-rating time was 1.22 × 10^9^.

From the perspective of the rating agent, the optimal model incorporated group and rating agent as fixed effects, with participants as the random effect (BF = 1.04 × 10^74^). The main effects of group and rating agent were significant, with the non-specific EFT group rated significantly lower than the specific EFT group (*b* = −0.117, *SE* = 0.031, *t* = −3.784, *p* < 0.001, 95%CI = [−0.18, −0.06], and the rater-rating scores were significantly lower than participants’ self-ratings (*b* = −0.730, *SE* = 0.029, *t* = −25.388, *p* < 0.001, 95%CI = [−0.79, −0.67]. The BFs for the group and the rating agent were 4.59 × 10^3^ and 1.36 × 10^74^, respectively. Across both LMMs, diagnostic checks indicated no convergence warnings or singular fit problems, low multicollinearity (all VIFs < 3), and no major violations of model assumptions.

### 3.3. Latent Profile Analysis

Table 2 presents the fit statistics for the latent profile models. Compared with the one-class solution, models with additional classes showed lower information criterion values, indicating improved fit. The two-class solution was supported by both the LMR-LRT and BLRT, suggesting that it fit the data better than the one-class model. However, the two-class solution appeared to provide only a relatively coarse representation of individual differences in EFT responsiveness. Although the three-class solution was not uniformly favored by all likelihood ratio tests, it converged properly and the best loglikelihood value was replicated across random starts, indicating satisfactory estimation stability. Moreover, the three-class model showed acceptable classification quality (entropy = 0.965), and its estimated class proportions (51.5%, 36.1%, and 12.4%) remained interpretable. While the four-class solution yielded further improvement in some fit indices, inspection of the profile trajectories suggested that it split the sample into conceptually overlapping subgroups, thereby reducing parsimony without adding meaningful substantive differentiation (Bauer & Curran, 2003). Taken together, these results indicated that the 3-class solution provided the best balance among statistical fit, convergence stability, class size, parsimony, and theoretical interpretability, and it was therefore retained for subsequent analyses.

### 3.4. Different Responsivity Patterns of Latent Classes

After an examination of participants’ self-rating scores in the two EFT groups (specific and non-specific EFT) across the different classes, we found that the three classes reflected distinct response patterns to EFT manipulation (see Table 3). Specifically, all latent classes exhibited significant self-ratings improvement after the EFT intervention, albeit with different magnitudes: minimal gains in Class 1 (*b* = −0.366, *SE* = 0.181, *t* = −2.025, *p* = 0.050, 95%CI = [−0.73, −0.01], Δ = 0.36), maximal enhancement in Class 2 (*b* = −3.414, *SE* = 0.241, *t* = −14.184, *p* < 0.001, 95%CI = [−3.91, −2.92], Δ = 3.42), and moderate progression in Class 3 (*b* = −2.000, *SE* = 0.258, *t* = −7.746, *p* < 0.001, 95%CI = [−2.58, −1.42], Δ = 2.00).

Different group distributions and responsive features across the three classes were unveiled after a deeper inspection. Class 1 (46.3% specific EFT vs. 53.7% non-specific EFT) was designated as *self-competent simulators*, as the relatively balanced distribution between groups suggested participants maintained competent self-initiated simulation abilities regardless of EFT specificity. Class 2 (62.1% specific EFT vs. 37.9% non-specific EFT) showed a clear predominance in the specific EFT condition, warranting their classification as *reactive simulators* due to their pronounced sensitivity to and reliance on structured guidance (i.e., specific EFT instructions). Conversely, Class 3 (30% specific EFT vs. 70% non-specific EFT) revealed a marked preference for the non-specific condition, earning the label *proactive simulators* as this distribution pattern reflected superior autonomous simulation capabilities requiring minimal external scaffolding.

To further examine whether the latent classification results reflected individual traits or state-dependent responses to the EFT manipulation, we performed a retrospective analysis of the LPA outcomes. First, we assessed the relationships between the LPA-derived groups and the VVIQ scores (reflecting stable imagery ability as a trait), as well as pre-imagery self-ratings (reflecting state-dependent responses to the EFT manipulation). The correlation analyses revealed that the LPA grouping was significantly related to pre-imagery self-ratings (*r* = −0.365, *p* < 0.001), but not with VVIQ scores (*r* = −0.087, *p* = 0.445). To further validate this relationship, we conducted a linear regression analysis with LPA grouping as the dependent variable and pre-imagery self-ratings as the predictor. Results confirmed that pre-imagery self-ratings significantly predicted latent groups, *F* (1, 79) = 11.996, *p* < 0.001, *R*^2^ = 0.133, β = −0.365, 95%CI = [−0.319, −0.086].

### 3.5. Task Performance Across Latent Classes

**Figure 3:**
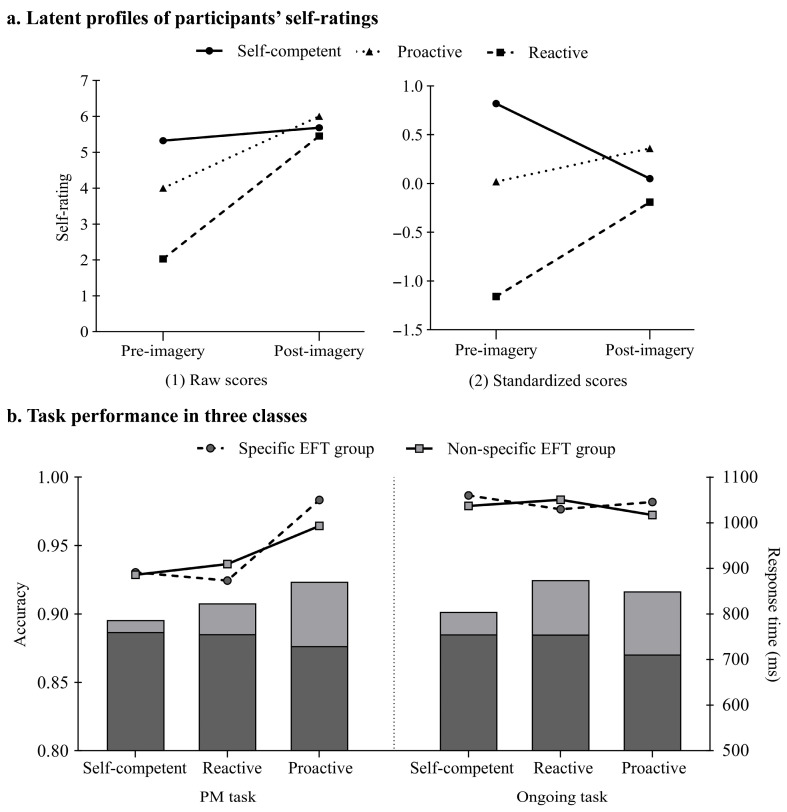
Behavior results of the three latent classes. (**a**) Delineates class-specific trajectories of participants’ self-rating scores: Panel (1) tracks raw score changes across two rating times for each class, while Panel (2) demonstrates the relative positional shifts in standardized scores for each class. (**b**) Illustrates dual task performance of the three latent classes. The line graph depicts the accuracy for each class, while the bar chart represents response times.

Considering the different number of participants in the three classes, the dual task performance data was weighted by class size. As shown in Table 3, the results revealed distinctive PM accuracies among the three classes (*F*(2, 79) = 4.357, *p* = 0.016, η^2^ = 0.089), with *proactive simulators* outperforming both *reactive* (*p* = 0.033) and *self-competent simulators* (*p* = 0.013). However, no between-class differences were observed for PM response time (*F*(2, 79) = 1.845, *p* = 0.165, η^2^ = 0.045), ongoing task accuracy (*F*(2, 79) = 0.564, *p* = 0.571, η^2^ = 0.016), or ongoing task response tine (*F*(2, 79) = 0.258, *p* = 0.773, η^2^ = 0.007). The descriptive results were illustrated in Figure 3.

## 4. Discussion

This study employed two EFT manipulation methods to examine their distinct effects on PM performance. Based on previous findings (Schacter & Addis, 2008; Madore & Schacter, 2016), we came up with two hypotheses. Hypothesis 1 suggested that both EFT methods could enhance PM performance, yet specific EFT would be more effective than non-specific EFT. Our results partially supported this assumption, revealing a notable finding that the specific EFT group demonstrated accelerated responses in PM tasks compared to the non-specific EFT group. Hypothesis 2 posited that the specific and non-specific EFT differ in encoding impacts and triggered automatic retrieval and strategic monitoring of PM respectively, which was reflected in the response time of the ongoing task (Shelton & Scullin, 2017). The results supported this hypothesis by displaying a more pronounced enhancement in response time to ongoing tasks in the specific EFT group.

Firstly, aligning with previous research (Altgassen et al., 2014, 2015; Cheng et al., 2021; Neroni et al., 2014; Cottini et al., 2021; Faustmann & Altgassen, 2024), this study revealed that EFT methods facilitated higher accuracy in PM tasks compared to the control group, demonstrating the effectiveness of EFT in enhancing PM performance. To wit, a successful future thinking simulation led to effective encoding of PM tasks, as evidenced by increased post-imagery self-rating scores of the two EFT groups. To be specific, EFT enhances the retrieval and recombination of episodic memory fragments, enabling the flexible construction of anticipated scenarios (Schacter & Addis, 2008). This constructive process allows individuals to pre-experience PM task execution, thereby strengthening memory traces of PM targets (external cues) and their association to the expected responses (Anderson et al., 2012; Cole et al., 2016; Paraskevaides et al., 2010). Such encoding mechanisms enhance participants’ ability to retrieve and execute PM intentions during PM tasks (Altgassen et al., 2016; Taylor et al., 1998; Taylor & Schneider, 1989), ultimately improving PM performance.

Secondly, our findings indicate that specific EFT results in superior PM performance compared to non-specific EFT, as evidenced by the shorter response time in the specific EFT group during PM tasks. This finding aligns with the *Constructive Retrieval Hypothesis*, which proposes that instructional guidance directing attention to specific episodic details during future event simulation serves to enhance both the episodic orientation (Madore & Schacter, 2016) and the quality of imagined scenarios (Tanguay et al., 2023). Such well-generated mental images contribute to the efficiency of information retrieval (Abel et al., 2024; Pylyshyn, 2002). Specifically, in the specific EFT group, researcher’s detailed instructions prompted participants to adopt a more specific orientation in the simulation of future scenarios, helping them to generate richer and more specific task-related imagery (as evidenced by higher post-imagery self-ratings). This detailed encoding aided in more efficient and meaningful association between PM cues and the future actions (Knäuper et al., 2009), enabling faster retrieval of PM intentions when detecting PM targets compared to the relatively superficial imagination of the non-specific EFT. As a result, participants can execute PM tasks more swiftly when prompted by PM cues, illustrating the effectiveness of specific EFT instruction in enhancing PM performance.

Moreover, it is noteworthy that a significant decrease in RTs of ongoing tasks was revealed in the specific rather than the non-specific EFT group, suggesting a potentially different main strategy was adopted by each group. According to the *Dynamic Multiprocess Framework*, one can balance well on two simultaneous tasks by realizing successful cognitive strategy switching during PM tasks (McDaniel & Einstein, 2000). Furthermore, stronger cue–action associations facilitate flexible shifts from strategic monitoring to automatic retrieval, thereby reducing cognitive load and minimizing time demands across tasks (Dagenais et al., 2016; Gonen-Yaacovi & Burgess, 2012). In the present study, we observed a slower speed in the ongoing task for the non-specific EFT group. It indicated that participants invested more cognitive resources in continuously monitoring PM cues, reflecting less efficient strategy switching and a heavier reliance on effortful monitoring. Conversely, the specific EFT group’s faster performance across both task types indicated a more effective strategy that alleviated PM-related cognitive demands, demonstrating adaptive shifts toward automatic retrieval.

These findings can be enlightened by *Temporal Construal Theory*, which states that future events are typically represented in abstract terms, with a focus on general perceptual features rather than specific incidental details (Trope & Liberman, 2003). However, the manipulation of the specific EFT group enabled participants to strengthen their representation of these details. Specifically, the specific EFT inductions enhanced participants’ encoding of contextual specifics by guiding them to transform abstract future scenarios into detailed, vivid simulations. This process likely strengthened cue–action associations and reinforced intention representation in retrospective memory (Potvin et al., 2011). The amplified connection in turn facilitated automatic retrieval, improving PM retention, initiation, and execution (Albiński et al., 2016; Galli, 2014; McDaniel et al., 2004), thereby preserving cognitive resources for parallel task processing. Consequently, the specific EFT group retrieved PM intentions more efficiently, with minimal disruption to ongoing task performance. By contrast, the non-specific EFT lacks this detail-enhancement process to further bolster the connection between PM targets and intention execution, which led to inefficient strategy shifts, heightened reliance on strategic monitoring (Pereira et al., 2012) and ultimately, greater cognitive resource depletion and impaired ongoing task performance.

Another key finding of this study is that three distinct classes of EFT response were identified through LPA—*self-competent*, *reactive*, and *proactive simulators*—each exhibiting unique responsiveness patterns to EFT manipulations. Retrospective analysis further revealed that these differences in responsiveness could be predicted by participants’ state-dependent cognitive characteristics. The *proactive simulators*, who showed relatively well-developed pre-existing cognitive schemas (as indicated by moderately high baseline self-ratings of future event imagery), seemed to be more responsive to non-specific EFT manipulation. One possible explanation is that these individuals may have partially formed mental representations of future events after the experiment instruction but prior to the EFT manipulation (Berntsen & Jacobsen, 2008). Therefore, they are potentially better able to efficiently retrieve and elaborate episodic fragments upon their pre-existing mental images with minimal external scaffolding. This cognitive advantage facilitated the construction of vivid images, as evidenced by their elevated post-imagery self-ratings. Such self-driven, detailed construction of future scenarios enhanced the encoding and maintenance of PM intentions (McFarland & Glisky, 2012; Spreng et al., 2018), ultimately improving PM task performance.

In contrast, the *reactive simulators* demonstrated the state of less processed pre-existing mental imagery, as evidenced by their lower baseline self-rating scores. Due to relatively simplistic nature of their initial mental representations, this class might rely more heavily on external scaffolding provided by the researchers to construct detailed event simulations. This aligns with the observation that a larger proportion of participants in this class were from the specific EFT group. However, the relative simplicity of their baseline representations may have constrained their ability to retrieve contextually rich details during guided imagery (Abel et al., 2024; Pylyshyn, 2002). Although post-intervention vividness ratings improved, they remained significantly lower than those of proactive simulators. This limitation in episodic detail retrieval during encoding likely hindered further PM performance gains (Ball & Bugg, 2018; Kuhlmann & Rummel, 2014; Scullin et al., 2018). The *self-competent simulators* exhibited no clear bias toward either EFT group, with near-equal distribution across conditions. Notably, these participants started the experiment with highly vivid, pre-formed mental imagery. Hence, neither EFT manipulation seemed to provide significant additional benefits to their imagery quality or PM performance. Taken together, these patterns suggest that the effectiveness of EFT manipulations may partly depend on individuals’ intrinsic future-thinking characteristics, although the tripartite profile structure identified herein should be interpreted as preliminary and requires replication in larger samples.

Collectively, the current findings advance our understanding of how varying levels of specificity in EFT differentially influence PM performance. Moreover, by using LPA, this study examined EFT subgroups based on participants’ state-dependent cognitive characteristics (i.e., pre-imagery self-ratings) and elucidated how these baseline states modulated responses to EFT manipulations. However, there are several limitations in the present study that should not be neglected. First, PM accuracy did not differ significantly between the two EFT groups. One possible explanation is that the formal test was administered after the PM simulations without a delay task, which might obscure potential advantages of specific EFT over non-specific EFT. A similar pattern was observed for response times, with no prominent differences found between the EFT groups and the control group. This null finding may stem from the use of a passive control group rather than an active reference task. Collectively, future research should incorporate both a delay task and an active control condition to better isolate potential differential effects between the two EFT groups. Second, while prior research typically finds PM tasks impair ongoing task performance due to cognitive resource competition (Heathcote et al., 2015; Loft & Remington, 2010), both EFT groups in our study showed improved ongoing task performance relative to the control group. This finding may reflect insufficient task difficulty in our paradigm, failing to adequately tax available cognitive resources. Subsequent studies should employ more demanding task designs to assess this resource allocation dynamic. Third, it has been proposed by *Dynamic Multiprocess Theory* that PM strategy selection operates as a dynamic process (Rummel & Kvavilashvili, 2023; Scullin et al., 2013). While our behavioral findings suggest differential effects of specific versus non-specific EFT methods on strategy adoption, the current study was limited to indirect inference of these dynamic selection processes. Future research could provide more direct evidence of strategy switching patterns under different EFT conditions by refining the paradigm or incorporating neurocognitive research to put forward a more profound understanding of the underlying mechanisms. Finally, the present sample consisted entirely of young adults, which limits the generalizability of the findings. Although previous work has begun to examine EFT effects in older adults and suggests that EFT may support PM in later life (Ma & Zhang, 2024; Terrett et al., 2016), it remains unclear whether the patterns observed here—particularly the differential effects of specific versus non-specific EFT on PM accuracy and response speed—would replicate in older populations under the current paradigm. Therefore, future studies should employ more age-diverse samples, especially older adults, to delineate the stability, boundary conditions, and age-related sensitivity of these EFT effects.

## 5. Conclusions

This study elucidated the differential effects of distinct EFT methods on PM abilities by comprehensively examining the impacts of specific and non-specific EFT within an experiment. By integrating accuracy and response time results, we demonstrated that both EFT methods exert a positive impact on PM performance, with specific EFT showing surpassing efficacy in reducing response times. These findings highlight the different cognitive strategies mainly adopted by each method in PM enhancement: specific EFT facilitates automatic retrieval, while non-specific EFT leads to more strategic monitoring. Additionally, the LPA results revealed individual differences in the responsiveness of EFT manipulations. This provides insight into the fact that individuals with different baseline imaginative features require different effective EFT methods to improve their PM abilities. Overall, this study advances our knowledge of the relationship between EFT and PM, illustrating how individual differences can shape the impact of EFT strategies and providing a foundational framework for tailoring EFT interventions to optimize PM performance.

## Figures and Tables

**Table 1:** Descriptive statistics results for three groups (*M* and *SD*).

	Specific EFT (*n* = 40)	Non-Specific EFT (*n* = 40)	Control (*n* = 33)	*F*/*t*	*p*	η^2^/*d*
Gender (male)	20	19	17	-	-	-
Age	18.60 (0.74)	18.83 (0.87)	18.85 (1.06)	0.907	0.407	0.016
VVIQ	3.67 (0.52)	3.56 (0.50)	0.97 (0.04)	1.204	0.304	0.021
Pre-imagery self-rating	3.80 (1.76)	4.13 (1.56)	4.15 (1.75)	0.518	0.597	0.009
Post-imagery self-rating	5.95 (0.82)	5.33 (1.07)	-	2.936	0.004	0.652
Rater-rating	12.32 (0.94)	10.84 (0.57)	-	8.549	<0.001	1.912

**Table 2:** Fit statistics of the latent profile analysis models.

Model	AIC	BIC	ABIC	*p*LMR-LRT	*p*BLRT	Entropy	*n* for Each Profile			
							1	2	3	4
One-class	482.895	492.423	479.810	–	–	–	80			
Two-class	446.163	462.837	440.764	<0.001	<0.001	0.961	29	51		
Three-class	444.511	468.331	436.798	0.4022	0.3077	0.965	41	29	10	
Four-class	369.481	400.447	359.453	0.6432	<0.001	1.000	10	19	41	10

Note: AIC = Akaike information criterion; BIC = Bayesian information criterion; ABIC = sample-size adjusted BIC; LMR = Lo–Mendell–Rubin; LRT = likelihood ratio test; BLRT = bootstrap likelihood ratio test.

**Table 3:** Descriptive statistics across the three latent classes (*M* and *SD*).

	Class 1	Class 2	Class 3	*F*	*p*	η^2^
VVIQ	3.82 (0.49)	3.54 (0.52)	3.71 (0.57)	1.845	0.165	0.048
Pre-imagery self-rating	5.32 (0.57)	2.03 (0.87)	4.00 (0.00)	202.590	<0.001	0.671
Post-imagery self-rating	5.68 (1.07)	5.45 (0.95)	6.00 (0.79)	2.305	0.107	0.052
Rater-rating	11.40 (1.14)	11.77 (0.19)	11.77 (0.27)	1.187	0.311	0.030
PM task ACC	0.93 (0.06)	0.93 (0.07)	0.97 (0.03)	4.357	0.016	0.089
PM task RT	774.17 (103.32)	781.19 (135.53)	828.36 (92.93)	1.845	0.165	0.045
Ongoing task ACC	0.98 (0.02)	0.98 (0.02)	0.98 (0.03)	0.564	0.571	0.016
Ongoing task RT	781.73 (132.04)	799.98 (140.58)	807.78 (34.66)	0.258	0.773	0.007

Note: Class 1: self-competent simulators; Class 2: reactive simulators; Class 3: proactive simulators. ACC = accuracy; RT = response time.

## Data Availability

The data that support the findings of this study are available from the corresponding author upon reasonable request.
